# Joint Estimation of Relative Risk for Dengue and Zika Infections, Colombia, 2015–2016

**DOI:** 10.3201/eid2506.180392

**Published:** 2019-06

**Authors:** Daniel Adyro Martínez-Bello, Antonio López-Quílez, Alexander Torres Prieto

**Affiliations:** University of Valencia, Valencia, Spain (D.A. Martínez-Bello, A. López-Quílez)**;**; Secretary of Health of the Department of Santander, Bucaramanga, Colombia (A. Torres Prieto)

**Keywords:** Risk maps, Bayesian models, multivariate risk models, conditional auto-regressive prior, Zika virus, dengue virus, viruses, Colombia

## Abstract

We jointly estimated relative risk for dengue and Zika virus disease (Zika) in Colombia, establishing the spatial association between them at the department and city levels for October 2015–December 2016. Cases of dengue and Zika were allocated to the 87 municipalities of 1 department and the 293 census sections of 1 city in Colombia. We fitted 8 hierarchical Bayesian Poisson joint models of relative risk for dengue and Zika, including area- and disease-specific random effects accounting for several spatial patterns of disease risk (clustered or uncorrelated heterogeneity) within and between both diseases. Most of the dengue and Zika high-risk municipalities varied in their risk distribution; those for Zika were in the northern part of the department and dengue in the southern to northeastern parts. At city level, spatially clustered patterns of dengue high-risk census sections indicated Zika high-risk areas. This information can be used to inform public health decision making.

Dengue and Zika virus disease (hereafter referred to as Zika) are infectious diseases caused by arboviruses in the family *Flaviviridae*. Dengue virus has 4 serotypes (1–4); serotypes 2 and 3 are associated with severe disease in persons with second dengue infections. Zika virus infection is associated with congenital malformations in babies born from women infected during pregnancy and with Guillain-Barré syndrome in infected adults (*1*).

Colombia is highly affected by vectorborne diseases. Villar et al. (*2*) reviewed the dengue epidemic in this country for 2000–2010 and reported an increasing epidemic trend for the period; outbreaks occurred in 2001, 2003, and 2010. In 2016, health authorities in Colombia reported >101,016 dengue cases that resulted in 289 deaths (*3*) and 9,799 Zika cases that were laboratory confirmed and 96,860 suspected Zika cases diagnosed by clinical signs (*4*).

For this study, we concentrated on the spatial patterns assessment of risk for dengue and Zika; in particular, we focused on the relative risk (RR) estimation for areal data by using hierarchical Bayesian models for these infections. RR represents the excess (or lack) of risk in a small area compared with the background risk. RR is mostly based on models and supported by Bayesian estimation methods (*5*). We used the following as study regions: the municipalities in the department of Santander, Colombia (1 of the departments where incidence of dengue and Zika for 2015–2016 was highest), and the city census sections belonging to the capital city of Santander (1 of the cities most affected by dengue and Zika for the same period).

Racloz et al. (*6*) and Louis et al. (*7*) reviewed the spatial patterns assessment of dengue risk; specifically for RR estimation of dengue, Ferreira and Schmidt (*8*) and Martínez-Bello et al. (*9*) estimated RR for dengue on a local spatial scale; and Restrepo et al. (*10*) and Martínez-Bello et al. (*11*) applied methods for the spatiotemporal assessment of dengue risk. Examples for the spatial patterns assessment of Zika risk run from merely descriptive methods to model-based approaches. For instance, in Colombia, descriptive risk maps associating Zika incidence rates with environmental and sociodemographic factors have been produced in the departments of Sucre (*12*), Tolima (*13*), Guajira (*14*), Santander, and Norte de Santander (*15*) and in the city of Pereira in the department of Risaralda (*16*). Model-based spatial patterns assessment of Zika risk have been developed for the 33 departments in Colombia by using Poisson models for the RR for Zika (*17*). The distribution of risk for Zika transmission among counties/districts in Guangdong Province, China, was assessed by using analytic hierarchy process models (*18*).

Zika, dengue, and chikungunya are also jointly studied by using the spatial patterns assessment of risk because the viruses share similar transmission routes (*Aedes* mosquitoes). On a large scale in Brazil, ecological studies have explored the risk factors for unusual spatial patterns of microcephaly, including dengue, Zika, and chikungunya data (*19*). Also estimated is the potential spatial risk for Zika and chikungunya according to socioenvironmental factors, estimating the size of the populations at risk for both diseases (*20*). On a small geographic scale, the risk factors for cocirculating arboviruses (dengue, Zika, chikungunya) at the community level have been evaluated (*21*).

In Colombia, Krystosik et al. (*22*) generated city-level risk maps of chikungunya, Zika, and dengue supporting vector-control strategies; Martínez-Bello et al. (*23*) estimated the RR for dengue and Zika by using spatiotemporal interaction effects models for 1 department and 1 city in Colombia. Riou et al. (*24*) assessed the spatial patterns of risk for the 2013 Zika and chikungunya outbreaks in the French Polynesia islands, and Funk et al. (*25*) jointly modeled Zika and dengue time series data from the Zika outbreak in Yap Island in the Pacific Ocean.

Our aim with this study was to jointly estimate the disease- and area-specific RRs for dengue and Zika by using hierarchical Bayesian joint models accounting for the spatial association between both diseases. We used data from the 2015–2016 Zika outbreak in Colombia and analyzed 2 levels of spatial data aggregation: the department level (disease counts aggregated per municipality) in the department of Santander and the city level (disease counts aggregated per census section) in the city of Bucaramanga (Santander).

## Materials and Methods

**Figure 1:**
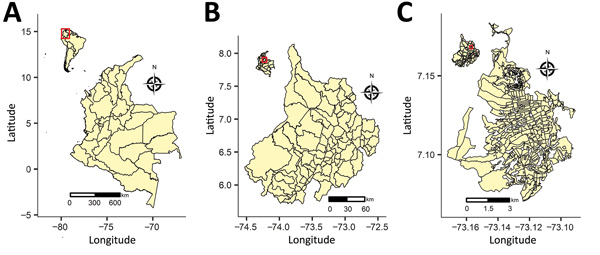
Location of the areas included in joint estimation of relative risk for dengue and Zika virus infections, Colombia, 2015–2016. A) Country of Colombia; inset shows location of Colombia in South America. B) Department of Santander; inset shows location of Santander in Colombia. C) City of Bucaramanga; inset shows location of Bucaramanga in Santander.

Colombia (Figure 1, panel A), population 48.7 million, comprises ≈1.14 million km^2^ divided into 33 administrative regions called departments, each 49.6–110,029.4 km^2^ (Table 1). The department of Santander (Figure 1, panel B) covers 30,642 km^2^, is divided into 87 municipalities, and has >2 million inhabitants. Santander is in the northeastern region of Colombia, and its administrative center, Bucaramanga (Figure 1, panel C), is in the northeastern region of the department. Bucaramanga covers 162 km^2^ (urban area 49.6 km^2^), is divided into 293 urban census sections, and according to the 2016 census has a population of 528,575. 

**Table 1:** Geographic divisions at national, department, and city levels, Colombia

Level	Division	Area, km^2^			
		Minimum	Mean	Maximum	Total
Colombia	33 departments	49.6	34,678.30	110,029.40	1,144,385
Santander	87 municipalities	18.8	352.20	3173.80	30,642
Bucaramanga	293 census sections	0.01	0.17	2.64	49.6

The Zika epidemic started on August 9, 2015 (*26*). Up to the first epidemiologic week of 2017, Colombia reported 106,659 Zika cases (219 cases/100,000 population) that were suspected by clinical signs and confirmed by laboratory. For the epidemiologic year 2016, the country reported 101,016 dengue cases (207.6 cases/100,000 population; Table 2). Since 2001, one of the departments with the highest dengue incidence in Colombia has been Santander (*2*); in 2016, its dengue incidence was the sixth highest among the 33 departments (331.63 cases/100,000 population) (*3*); in 2015–2016, its Zika incidence was the seventh highest (493.09 cases/100,000 population) (*4*). The incidence rate for Zika in Santander was higher than the national average, accounting for 9.76% of the total cases in Colombia (*3*). With regard to dengue, in 2016 Santander reported 6.67% of the total cases for Colombia (Table 2). Within the department, 35.52% of Zika cases (692.81/100,000) and 35.62% of dengue cases (467.3/100,000) were reported in Bucaramanga (Table 2).

**Table 2:** Zika and dengue suspected and confirmed cases reported in Colombia, department of Santander, and city of Bucaramanga, 2015–2016

Disease, level	Suspected*	Confirmed†	Total‡	Incidence rate§	Population × 1,000
Zika					
Colombia¶	96,860	9,799	106,659	219.0	48,654
Santander#	9,420	547	9,967	493.1	2,090
Bucaramanga**	No data	No data	3,662	692.8	529
Dengue					
Colombia††	100,117	899	101,016	207.6	48,654
Santander††	No data	No data	6934	331.6	2,090
Bucaramanga**	No data	No data	2470	467.3	529

*Based on clinical signs.  †Laboratory confirmed. ‡Suspected cases with laboratory confirmation. §(Total no. cases/population × 1,000) × 100,000. ¶August 9, 2015–January 5, 2017 (*3*). #Epidemiologic week 45 of 2015 to epidemiologic week 52 of 2016 (*3*). **Epidemiologic week 45 of 2015 to epidemiologic week 52 of 2016 (this study). ††Epidemiologic weeks 1–52, 2016 (*2*).

### Department-Level Data

We aggregated cases of dengue and Zika for the study period (from epidemiologic week 42 in 2015 through epidemiologic week 52 in 2016) obtained from SIVIGILA (the public health surveillance system of Colombia) in the 87 municipalities of Santander by using cartography from the National Geostatistical Framework (*27*). The case definitions for dengue and Zika correspond to codes 210 (dengue), 220 (severe dengue), 580 (dengue mortality), and 895 (Zika), obtained from the SIVIGILA protocols for dengue (*28*) and Zika (*29*). We calculated expected values of dengue and Zika by municipality by using 2016 population projections provided by the Colombia Administrative Department of National Statistics. We first calculated incidence rates for dengue and Zika for 5-year patient age groups and for sex on the basis of observed cases recorded during the study period. We then multiplied these age- and sex-specific incidence rates to the population structure of each municipality, generating disease-specific expected values for both diseases. Last, we aggregated the age- and sex-specific expected values per municipality of dengue and Zika to obtain standardized expected counts per municipality for dengue and Zika.

### City-Level Data

Cases of dengue and Zika, notified in Bucaramanga and obtained from SIVIGILA for the study period, were geocoded and allocated to 293 census sections in the city (*27*). A census section is a cartographic unit comprising 1–9 census blocks, consisting of built or unbuilt lots bounded by public roads or pedestrian walkways (*27*). We calculated expected values of dengue and Zika by census sections by using a similar method to the one applied at the department level. Using the 2016 population projections provided by the Colombia statistical office, we calculated incidence rates for dengue and Zika by 5-year patient age groups and sex for the study period. Next, we multiplied the incidence rates for the population structure (by sex and age group) of each census section obtained from the Colombia Census and aggregated the age- and sex-specific expected counts per census section to obtain the standardized expected values of dengue and Zika per census section.

### Joint Models for Estimating Relative Risk

We assumed that the observed counts of dengue or Zika aggregated by area (87 municipalities at the department level or 293 census sections at the city level) follow a Poisson distribution, with mean parameter equal to the product of the disease- and area-specific expected values and RR. The logarithm of the RR is the additive result of disease- and area-specific random effects accounting for the uncorrelated and clustered spatial patterns of risk and possibly covariates (*30*). Random effects for spatially clustered patterns of risk are unobserved variables recovering risk autocorrelation between adjacent areas, indicating that risk in one area is highly associated with risk in neighboring areas. The lack of spatial risk autocorrelation is accounted for by spatial uncorrelated random effects (*5**,**31*).

A joint model of RR for dengue and Zika defines a structure for the nature of the spatial association between the diseases (*5*). To that end, we fitted 8 Bayesian Poisson models (*5**,**31*) of joint estimation of RR to the dengue and Zika data at the department and city levels. Formulation of the statistical model is available in the Appendix. Here we describe the spatial patterns of risk for every joint model.

**Figure 2:**
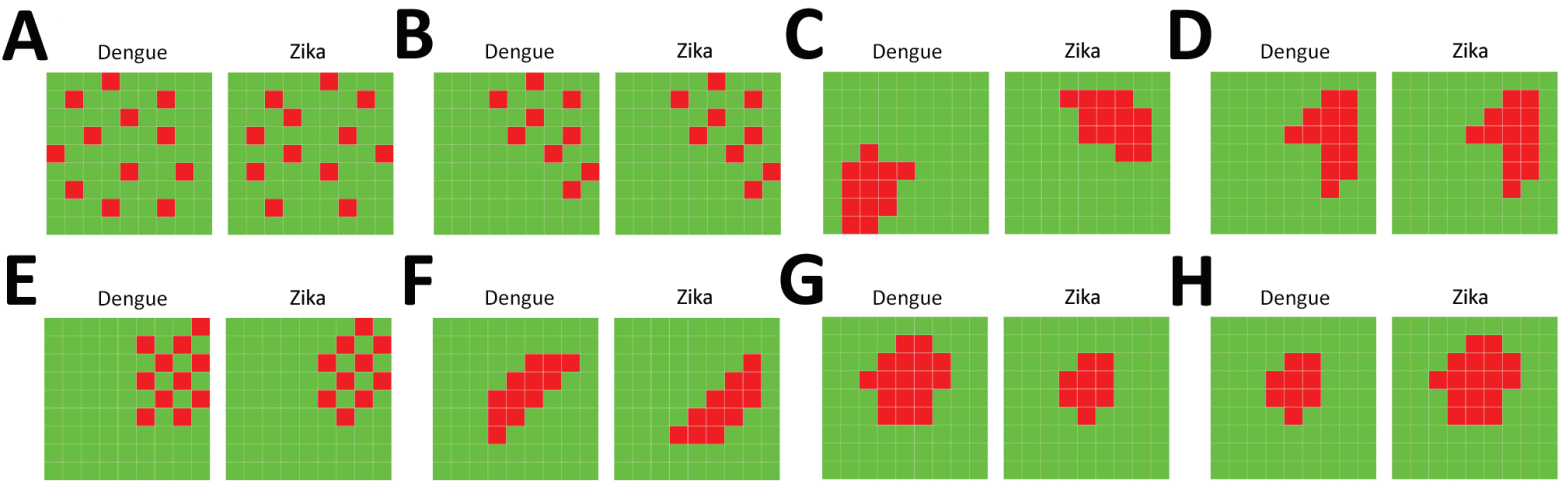
Schematic representation of the spatial patterns of dengue and Zika risk revealed by the joint models of relative risk models, Colombia, 2015–2016. A) Model 1; B) model 2; C) model 3; D) model 4; E) model 5; F) model 6; G) model 7; H) model 8. For a set of small areas, high-risk areas are represented in red and low-risk areas are represented in green, depicting several patterns that could or could not be shared for both diseases in the same geographic area.

Model 1 contains area- and disease-specific random effects capturing uncorrelated spatial patterns of dengue and Zika risk. Choosing this model implies that dengue and Zika high-risk areas are not associated with each other and show no clustering (Figure 2, panel A).

Model 2 incorporates area- and disease-specific random effects capturing uncorrelated spatial patterns of dengue and Zika risk, where the random effects of both diseases are spatially associated. Model 2 assumes that the random effects for uncorrelated spatial patterns are linearly associated for both diseases; thus, dengue and Zika high-risk areas occur at the same position (Figure 2, panel B).

Model 3 contains area- and disease-specific random effects representing spatially clustered patterns of dengue and Zika risk, where the random effects are not associated between diseases. In this model, dengue high-risk areas are clustered and Zika high-risk areas are clustered, but dengue and Zika high-risk clustered areas are not spatially associated (Figure 2, panel C).

Model 4 contains area- and disease-specific random effects revealing spatially clustered patterns of dengue and Zika risk, where the random effects are linearly associated between diseases. In this model, dengue and Zika high-risk areas are spatially associated; thus, there are spatially clustered patterns of dengue and Zika high-risk areas at the same spatial locations (Figure 2, panel D).

Model 5 incorporates area- and disease-specific random effects revealing uncorrelated spatial patterns of dengue and Zika risk, and the spatial association between both diseases is modeled by using shared components of clustered spatial patterns of dengue and Zika risk. In this model, dengue high-risk areas are next to Zika high-risk areas, but dengue high-risk areas are not clustered and Zika high-risk areas are not clustered (Figure 2, panel E).

Model 6 incorporates area- and disease-specific random effects revealing spatially clustered patterns of dengue and Zika high risk, and the spatial association between both diseases is accounted for by using shared random effects of spatially clustered patterns of dengue and Zika high risk. In this model, spatially clustered patterns of dengue high-risk areas are adjacent to spatially clustered patterns of Zika high-risk areas (Figure 2, panel F).

Model 7 contains area- and disease-specific random effects accounting for spatially clustered patterns of Zika high risk conditioned on random effects for spatially clustered patterns of dengue high risk. Thus, dengue high-risk areas are determinants of the presence of Zika high-risk areas (Figure 2, panel G).

Model 8 contains area- and disease-specific random effects accounting for spatially clustered patterns of dengue high risk conditioned on random effects for spatially clustered patterns of Zika high risk. Thus, Zika high-risk areas are determinants of the presence of dengue high-risk areas (Figure 2, panel H).

We summarized the association structure of the joint models of RR (Table 3); every model captures the nature of the spatial association between dengue and Zika risk. We fitted the Bayesian Poisson models by applying Markov chain Monte Carlo simulations and using WinBUGS 1.4 software (*32*) for Bayesian analysis. We selected noninformative prior distributions for the parameters and hyperparameters; model specifications are presented in the Appendix. Every model was fitted by using 100,000 burn-in iterations, 10,000 iterations for inferences, a thinning of 10, three chains, and 1,000 iterations per chain for final inferences. For model selection, we used the deviance information criterion (DIC) (*33*), for which the lowest DIC estimates the model with the best predictions subject to the DIC difference between the model with minimum DIC and the next model being >5, as substantial evidence in favor of the first model with respect to the second (*34*).

**Table 3:** Association structure assumed by relative risk models fitted to department- and city-level dengue and Zika data for the 2015–2016 Zika virus disease outbreak, Colombia

Model no.	Spatially structured association between dengue and Zika high-risk areas	Joint association between dengue and Zika high-risk areas
1	No	No
2	No	Yes, linear
3	Yes	No
4	Yes	Yes, linear
5	No	Spatially structured shared component
6	Yes	Spatially structured shared component
7	Yes	Zika risk conditioned by dengue risk
8	Yes	Dengue risk conditioned by Zika risk

## Results

**Figure 3:**
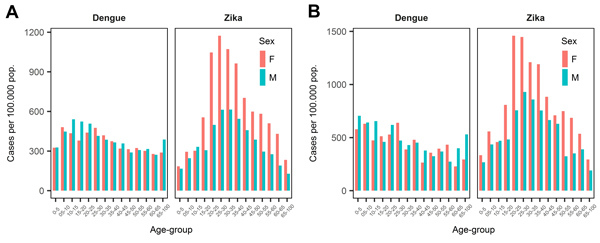
Incidence rate of dengue and Zika per 100,000 population by age group and sex, Colombia, 2015–2016. A) Department of Santander; B) city of Bucaramanga.

For the Santander department, the analysis included 10,051 Zika patients (63.1% female, 36.9% male) and 7,891 dengue patients (48.6% female, 51.4% male). The analysis of Bucaramanga city included 3,662 Zika patients (61.2% female, 38.8% male) and 2,470 dengue patients (49.3% female, 50.7% male). Figure 3 shows the age- and sex-specific incidence rates for dengue and Zika in Santander (Figure 3, panel A) and Bucaramanga (Figure 3, panel B). At the department and city levels, reported Zika cases were consistently higher among female patients in the 10–14 and 55–59 year age groups, and reported dengue cases were highest among patients of both sexes in the 5–9 and 20–24 year age groups. The age- and sex-specific incidence rates were slightly higher at the city level than the department level.

**Figure 4:**
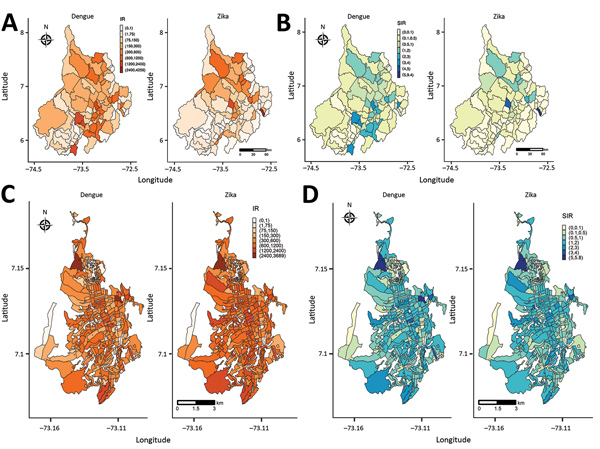
IRs and SIRs for dengue and Zika virus disease, Colombia, 2015–2016. A) Santander IR; B) Santander SIR; C) Bucaramanga IR; D) Bucaramanga SIR. IR, incidence rate; SIR, standardized incidence rate.

We mapped the incidence rates (cases/100,000 population) and the standardized incidence ratios (observed values/expected values) for dengue and Zika (Figure 4). At the department level, the incidence rate for Zika was 0–3,688 cases/100,000 population; for dengue, 0–4,285 cases/100,000 population (Figure 4, panel A). At the department level, the incidence rate for dengue was highest in the southeastern municipalities; for Zika, in the northeastern municipalities. The standardized incidence ratios for dengue and Zika at the department level follow the findings of the incidence rate map; however, the standardized incidence ratio accounts for the expected number of cases, so some areas with a high observed incidence rate do not show a high standardized incidence ratio (Figure 4, panel B). We determined heterogeneous incidence rates for dengue and Zika at the city level (Figure 4, panel C); some high incidence rate census sections are in the northern and central areas of the city. We also determined the standardized incidence ratio per census section (Figure 4, panel D); as before, the standardized incidence ratio map shows the findings of the incidence rate, smoothing the geographic incidence estimates by census sections in Bucaramanga.

We compiled the results of the selection statistics for the joint models of RR at the department level (Table 4). Based on the lowest DIC, model 5 (disease-specific random effects for uncorrelated spatial patterns of risk and shared random effects of spatially clustered patterns for both diseases) is the selected model (deviance 808.3, DIC 942.9) for Santander; at the city level (Table 5), model 7 (Zika risk distribution conditioned by dengue risk) is the selected final model (deviance 2,869.7, DIC 3,119.3) for Bucaramanga. We included the DIC differences between models, showing that after the final selected model, models 7 and 8 were the closest models at the department level; models 8 and 4, at the city level (Tables 4, 5).

**Table 4:** Selection statistics from the joint models of relative risk for dengue and Zika, department of Santander, Colombia, 2015–2016*

Model no.	Deviance	Parameter	DIC	Δ-DIC						
				M2	M3	M4	M5	M6	M7	M8
1	810.9	144.6	955.5	1.3	359.9	321.6	12.6	330.7	5.5	4.1
2	814.2	140.0	954.2		361.2	322.9	11.3	332	4.2	2.8
3	991.4	324.0	1315.4			38.3	372.5	29.2	365.4	364
4	976.8	300.4	1277.1				334.2	9.1	327.1	325.7
5	808.3	134.6	942.9					343.3	7.1	8.5
6	987.3	299.0	1286.2						336.2	334.8
7	810.4	139.6	950.0							1.4
8	811.7	139.7	951.4							

*Blank cells indicate no data available. DIC, deviance information criterion; M, model; Δ-DIC, DIC difference.

**Table 5:** Selection statistics from the joint models of relative risk for dengue and Zika, city of Bucaramanga, Colombia, 2015–2016*

Model no.	Deviance	Parameter	DIC	Δ-DIC						
				M2	M3	M4	M5	M6	M7	M8
1	2870.1	364.9	3232.1	99.8	22.3	101.3	97.9	100	112.8	102.9
2	2853.3	279.0	3132.3		122.1	1.5	1.9	0.2	13	3.1
3	2922.4	331.9	3254.4			123.6	120.2	122.3	135.1	125.2
4	2884.2	246.6	3130.8				3.4	1.3	11.5	1.6
5	2856.8	277.4	3134.2					2.1	14.9	5
6	2888.9	243.3	3132.1						12.8	2.9
7	2869.7	249.6	3119.3							9.9
8	2874.2	255.0	3129.2							
*Blank cells indicate no data available. DIC, deviance information criterion; M, model; Δ-DIC, DIC difference.										

**Figure 5:**
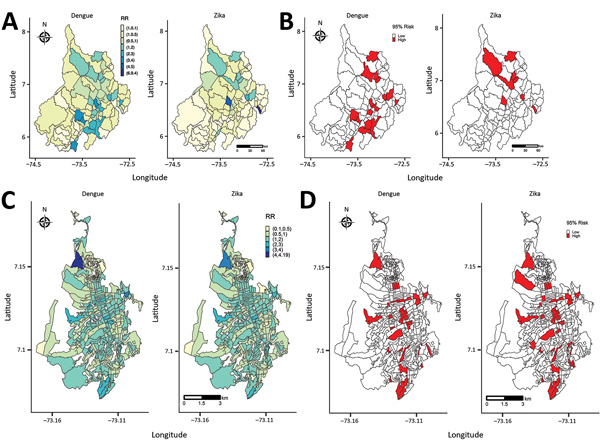
Posterior mean RR and 95% credible interval (CrI) of RR >1 (95% CrI RR >1), from model 5 for the department of Santander and from model 7 for the city of Bucaramanga, Colombia, 2015–2016. A) Posterior mean RR for Santander; B) 95% CrI RR >1 for Santander; C) posterior mean RR for Bucaramanga; D) 95% CrI RR >1 for Bucaramanga. RR, relative risk.

Figure 5 displays the posterior mean RR and the lower bound of the 95% credible interval (CrI) of RR >1 (95% CrI RR >1) of dengue and Zika obtained from the selected final models in Tables 4 and Tables 5. In Santander, the posterior mean RR (Figure 5, panel A) shows the standardized incidence ratio pattern displayed in Figure 4, panel A; as a byproduct of the modeling process, Figure 5, panel B, shows the municipalities with 95% probability of RR higher than that for the other municipalities. Most of the dengue and Zika high-risk municipalities differ in risk distribution: Zika high-risk municipalities are in the northern part of the department, and dengue high-risk municipalities are in the southern to northeastern parts. For Bucaramanga, the dengue and Zika posterior mean RR maps revealed the nonclustered risk pattern of the diseases (Figure 5, panel C), also displayed by the standardized incidence ratio map in Figure 4, panel C. However, the model shrinks the posterior mean RR, capturing the close association between Zika and dengue high-risk distribution per census sections. We identified the census sections with 95% probability of RR being higher than in the other areas (Figure 5, panel D), showing dengue high-risk census sections associated with Zika high-risk census sections at the city level.

## Discussion

Our study illustrates the joint estimation of RR for dengue and Zika at the department and city levels in Colombia. A battery of joint models of RR captured the spatial association between the diseases during the 2015–2016 Zika outbreak. The model selection process was based on DIC statistics; and we assessed the model’s goodness-of-fit by using residual analysis, fitted-observed scatter plots, and the posterior predictive checks of overdispersion recovery (Appendix).

At the department level, the selected model 5 reveals spatially clustered patterns of high-risk municipalities for both diseases, while keeping disease- and area-specific uncorrelated spatial patterns of high-risk municipalities, precluding selection of other feasible but not optimal models, such as models 7 and 8, containing area- and disease-specific spatially clustered patterns of high risk. The selected model shows that dengue- and Zika-specific high-risk municipalities are not clustered but that dengue high-risk municipalities are next to Zika high-risk municipalities.

At the city level, the selected model 7 contains conditional random effects for spatially clustered patterns of high-risk census sections, where Zika high-risk census sections are conditioned on dengue high-risk census sections; thus, Zika high-risk census sections are highly associated with dengue high-risk census sections. Models 8 and 4 were other feasible but not optimal options; although both models reveal the spatially clustered distribution of risk, the spatial data distributions do not support strong spatial association of dengue high-risk areas at the same locations as Zika high-risk areas. Model 7 shows that dengue or Zika high-risk areas are spatially clustered; dengue and Zika high-risk census sections are near other high-risk census sections. Thus, at the city level, clustered census sections display favorable conditions for transmission of both diseases, justifying a deeper study of environmental, infrastructural, and socioeconomic conditions associated with dengue and Zika high risk in those areas.

Model-based estimates of posterior means and lower bounds of CrIs of RR were represented in risk maps. These maps identified areas with a given risk probability and highlighted municipalities or census sections with high probable risk for dengue or Zika.

The area- and disease-specific risk estimates from the joint models accounting for the spatial association of both diseases (models 5 [department level] and model 7 [city level]) were more accurate than the estimates recovered ignoring the association of both diseases. It would be useful to compare risk estimates from joint models, with estimates ignoring the association structure of cocirculating arboviral diseases. For instance, Krystosik et al. (*22*) estimated the combined risk for dengue, Zika, and chikungunya but did not provide disease-specific risk estimates; Costa et al. (*35*) found associations in the spatial distribution of dengue, Zika, and chikungunya but did not use the information on the diseases’ association to improve the accuracy of the risk estimates. The special characteristics of the joint modeling of cocirculating arboviruses provide an epidemiologic tool for transmission control and disease mitigation (*36*).

Our study has some limitations. Although notification of dengue and Zika is compulsory in Colombia, a large proportion of reported cases are diagnosed by clinical signs and not laboratory confirmed. Because we used suspected and confirmed dengue and Zika cases, some cases included in the study may have been misdiagnosed. Another potential source of study bias is underreporting, as shown by Romero-Vega et al. (*37*) for dengue in Colombia; the National Health Institute of Colombia calculated an underreporting rate of 49% for Zika in a high-incidence town in Colombia (*38*). However, we used the same data that the Colombia authorities use to generate public health information for making decisions about controlling and preventing activities of public and private health institutions, extending the current information produced by the public health surveillance system, supporting surveillance activities for Zika and dengue, providing information for monitoring the geographic distribution of both diseases, and characterizing spatially the disease distribution in the population (*39*).

Determinants of dengue and Zika risk as covariates within the joint models (e.g., using datasets such as the ecological and environmental spatial dataset developed by Siraj et al. [*40*] for Colombia) remain to be elucidated. Other joint models available in the spatial analysis literature should be tested, jointly modeling other arboviral diseases such as chikungunya together with dengue and Zika and including joint models of RR of dengue and Zika within real-time surveillance platforms (*41*). 

In summary, our method for mapping cocirculating dengue and Zika provides a tool for describing disease distribution, based on the epidemiologic complexities of both diseases. Information on the association between diseases is of value, especially in areas where multiple arboviruses cocirculate, and can be used to improve inferences and interpretations and thus contribute to informed public health decision making.

Appendix Statistical formulation used for joint estimation of relative risk for dengue and Zika virus infections, Colombia, 2015–2016.
