# Supplementary material for: Joint Estimation of Relative Risk for Dengue and Zika Infections, Colombia, 2015–2016
Source: Emerg Infect Dis. 2019 Jun;25(6):1118–26. doi: 10.3201/eid2506.180392 (PMC6537708; doi:10.3201/eid2506.180392)
Supplement: Appendix — Statistical formulation used for joint estimation of relative risk for dengue and Zika virus infections, Colombia, 2015–2016. [file 18-0392-Techapp-s1.pdf]

Article DOI: https://doi.org/10.3201/eid2506.180392

Estimating Relative Risk for Dengue and Zika Virus Infections, Colombia, 2015–2016

Appendix

Supplementary Material

Together with this supplementary material, the reader can use the .txt files located at https://github.com/danieladyro/SupplementaryMaterialEID, containing the WinBUGS 1.4 code, data, and initial values necessary to reproduce the results shown in the manuscript as a result of fitting the joint models of relative risk 1 to 8 for the department of Santander and the city of Bucaramanga.

Statistical formulation of the joint models of relative risk

Let us assume the observed counts O_{ij} of dengue or Zika virus disease (ZVD) are Poisson distributed with mean parameter (μ_{ij}) where *i* is the aggregation area (*i* = 1,..,*n*, and *n* = 87 municipalities at departmental level; or *n* = 293 census section for the municipal level), and *j* is the disease (*j* = 1,…,*p*, and *p* = 1 for Dengue or *p* = 2 for ZVD), then,

$$O_{ij} \sim Poisson(\mu_{ij})$$
$$\mu_{ij} = E_{ij} \times r_{ij}$$
$$r_{ij} = \exp(\lambda_{ij})$$
$$\lambda_{ij} = \alpha_j + \phi_{ij}$$

Thus, the mean parameter μ_{ij} is equal to the product of the expected values E_{ij} and the relative risk r_{ij} with λ_{ij} linear predictor. The λ_{ij} linear predictor includes an α_j intercept for every disease, and the ϕ_{ij} parameter which could be spatially clustered or uncorrelated random effects or covariates. Spatially clustered random effects are unobserved variables recovering spatial clustered patterns of risk, or the fact that the one area’s risk is highly associated with the neighboring areas. The lack of spatial association is accounted by the spatially uncorrelated random effects (1).

All the models shown in the following rely in different formulations for the ϕ_{ij} parameter, because every formulation accounts for a different association structure between the disease-specific ϕ_{ij} parameters. Models 1 and 2 in Appendix Table 1 contain spatially uncorrelated random effects linearly un-associated (Model 1) or associated (Model 2) between both diseases. Models 3 and 4 in Appendix Table 2 contain spatially clustered random effects linearly associated (Model 4) or not (Model 3). Models 5 and 6 in Appendix Table 3 contain disease-specific spatially uncorrelated (Model 5) and clustered (Model 6) random effects including a spatially clustered shared-component for both diseases. Models 7 and 8 in Appendix Table 4 fit spatially clustered random effects of dengue (or ZVD) conditioned on spatially clustered random effects of ZVD (or dengue).

In Appendix Figure 1, we can appreciate the different spatial patterns of risk that each joint model accounts.

Calculating expected values of dengue and ZVD

The joint models of relative risk require the disease- and area-specific observed counts and the expected counts. Expected counts are obtained by external or internal standardization. External standardization requires incidence rates (cases per 100,000 people) by age groups and sex obtained from a standard or reference population, while internal standardization uses incidence rates obtained from the same data. In the manuscript, we used internal standardization to compute the expected values. The incidence rate is computed by

$$IR_{pq} = \frac{Cases_{pq}}{Population_{pq}} \times 100{,}000$$

where $\boldsymbol{IR_{pq}}$ is the incidence rate in age groups p = 1,…,P, (1 = [0; 5); 2 = [5; 10),…, 14 = [65; 100)) and sex q = 1, 2, (1=female, 2= male); $\boldsymbol{Cases_{pq}}$ are the total number of ZVD or dengue cases; and $\boldsymbol{Population_{pq}}$ is the total population in age group p and sex q for the departmental or city level over the complete study period, as obtained from the 2016 projected population of the Colombian census 2005. Using the $\boldsymbol{IR_{pq}}$, the expected values of dengue or ZVD per municipality at departmental level, or census section at city level were calculated using:

$$E_i = \sum_{p=1}^{P}\sum_{q=1}^{2}\left(IR_{pq} \times Population_{ipq}\right)$$

where $\boldsymbol{E_i}$ is the expected value in small area *i*, and $\boldsymbol{Population_{ipq}}$ is the census population in small area *i*, age group *p* and sex *q*. At the end, we remain with four sets of expected values of dengue and ZVD: Santander's ZVD and dengue expected values, and Bucaramanga's ZVD and dengue expected values.

Using the observed and expected values of dengue and ZVD, a raw estimator of risk is the ratio between the observed and the expected values of dengue and ZVD per area *i* which is the standardized incidence ratio (SIR),

$$SIR_i = \frac{Observed_i}{Expected_i}$$

The standardized incidence ratio are plotted in maps in the manuscript, as a descriptive step of modeling relative risk at departmental and city level.

Diagnostic measures for the joint models of relative risk

For model diagnosis we use posterior predictive checks, residual histograms, and scatter plots and Spearman correlation coefficient of the observed versus the fitted counts. To assess whether the model recovers the over dispersion observed in the data we compare the ratio $C_z = Var(Z)/\bar{Z}$ based on sampled new data with the corresponding ratio C_y for the observed counts (2). This check was done at each iteration and a satisfactory model will have C_z exceeding C_y about 50% of the time.

$$P_c = E\left[\Pr(C_z > C_y|y)\right]$$

Values of $\widehat{P_c}$ near 0 or 1 (above 0.9 or below 0.1) indicate discrepancy between the observations and the model, while values close to 0.5 mean that the observed data and the fitted data sampled from the model are closely comparable in terms of the over dispersion function. Then, Appendix Table 5 shows the predictive checks on over dispersion from the joint models 1 to 8 in Santander and Bucaramanga. Following the decision criteria, all the joint models for the department of Santander recovers the over dispersed data, because the predictive checks show an

average probability of $\widehat{P_c}$ = 0.50, while the predictive checks for the city of Bucaramanga show some models with acceptable posterior predictive checks for over dispersion recovery (Models 1 to 7 for dengue, and Models 1, 3 and 8 for ZVD), and with acceptable but close to the boundary probabilities for Model 8 for dengue and Models 2, 4, 5, 6, and 7 for ZVD. The conclusion is that in the city of Bucaramanga all models clearly recovered the over dispersion, however ZVD data were more difficult to fit than dengue data, and in the department of Santander, all models recovered over dispersion very well. Figure 2 displays the residual histograms of the posterior mean of the fitted values in Santander (A) and Bucaramanga (B). At departmental level, model residuals shrinks to zero, and the residual boundaries are -1.5 to 1. In the city of Bucaramanga, model residuals are more dispersed than residuals in Santander, and the residual boundaries are from -2.5 to 5.0. From the residual examination, similar to the predictive checks in over dispersion, we observe that the joint models fitted the data worse for the city level than for the departmental level. The association between the posterior mean of the fitted values and the observed counts of dengue or ZVD is accounted using scatter plots of fitted versus observed and the assessment of the Spearman correlation coefficient. For the department of Santander, the association of fitted and observed values in Figure 3 (A) in Bucaramanga and (B) in Santander reveal that all models generate fitted values very close to the observed counts, which is ratified by the near to one Spearman correlation coefficients from Appendix Table 6. For the city of Bucaramanga, we observe that there are some variability in the association fitted- observed, so the models are missing something in following the observed counts, which is confirmed by the Spearman correlation coefficients near to 0.950 for most of the models. As an overall conclusion, joint models work very well by predicting the observed counts of dengue and ZVD, recovering the data over dispersion, and reducing the dispersion revealed in the residuals for the departmental level, although the performance could be improved at the city level. Reasons for the lower performance of the joint models at city level could be explained by the need to include other parameters in the linear predictor, for instance the inclusion of correlated as well uncorrelated heterogeneity parameters at the same time, or perhaps to difficulties of the model to fit high number of areas (293 at city level compared with 87 areas at departmental level). Although the lower performance of the joint models at city level is not an ideal predictive feature, we have observed that the linear association fitted-observed counts is greater than 0.92, which is not so bad, so we can proceed to make model selection based on the DIC.

References

1. Banerjee S, Carlin BP, Gelfand AE. Hierarchical Modeling and Analysis for Spatial Data, 2nd Edition. Chapman & Hall/CRC Monographs on Statistics & Applied Probability. Boca Raton (FL): Chapman & Hall; 2014.

2. Congdon P. Bayesian Models for Categorical Data. Wiley Series in Probability and Statistics. New York: John Wiley & Sons; 2005.

Appendix Table 1. Statistical formulation of joint models of relative risk 1 and 2.

Model 1	Model 2
This model contains disease-specific independent and identically distributed (IID) Normal spatially uncorrelated random effects for dengue and ZVD.	This model contains disease-specific independent and identically distributed (IID) Normal spatially uncorrelated random effects linearly correlated for both diseases.
$O_{ij} \sim \text{Poisson}(\mu_{ij})$<br>$r_{ij} = \alpha_j + \phi_{ij}$<br>$\log(\mu_{ij}) = \log(E_{ij}) + \alpha_j + \phi_{ij}$<br>$\boldsymbol{\phi}_j \sim \text{Normal}(\mathbf{0}, \sigma^2_{\phi_j}\boldsymbol{I})$<br>$\alpha_j \sim \text{Normal}(0,1000)$<br>$\frac{1}{\sigma^2_{\phi_j}} \sim \text{Gamma}(0.01,0.01)$	$O_{ij} \sim \text{Poisson}(\mu_{ij})$<br>$r_{ij} = \alpha_j + \phi_{ij}$<br>$\log(\mu_{ij}) = \log(E_{ij}) + \alpha_j + \phi_{ij}$<br>$\begin{bmatrix}\boldsymbol{\phi}_1 \\ \boldsymbol{\phi}_2\end{bmatrix} \sim \text{Normal}(\mathbf{0}, \boldsymbol{\Sigma} \otimes \boldsymbol{I})$<br>$\boldsymbol{\Sigma} = \begin{bmatrix}\sigma^2_{\phi_1} & \rho\sigma_{\phi_1}\sigma_{\phi_2} \\ \rho\sigma_{\phi_1}\sigma_{\phi_2} & \sigma^2_{\phi_2}\end{bmatrix}$<br>$\boldsymbol{\Sigma}^{-1} \sim \text{Wishart}(\boldsymbol{R}, 2)$<br>$\boldsymbol{R} = \begin{bmatrix}1/5 & 0 \\ 0 & 1/5\end{bmatrix}$<br>$\alpha_j \sim \text{Normal}(0,1000)$
The $\boldsymbol{\phi}_j$ are spatially uncorrelated random effects, the α_j are intercepts, $\sigma^2_{\phi_j}$ are variance parameters of the $\boldsymbol{\phi}_j$, and $\boldsymbol{I}$ is an $n \times n$ identity matrix, and r_{ij} is the relative risk in area *i* and disease *j*.	$\boldsymbol{\Sigma}$ is an $n \times n$ variance-covariance matrix accounting for the linear association of the spatially uncorrelated random effects $\boldsymbol{\phi}_j$, $\otimes$ corresponds to the Kronecker product of two matrices, **R** is the parameter matrix for the Wishart distribution, and the other parameters similar to Model 1.

Appendix Table 2. Statistical formulation of joint models of relative risk 3 and 4

<table>
<tr><td>Model 3</td><td>Model 4</td></tr>
<tr><td>This model accommodates conditionally autoregressive (CAR) Normal spatially clustered random effects for every disease, which are not linearly associated.</td><td>This model contains CAR Normal spatially clustered random effects linearly associated for both diseases.</td></tr>
<tr><td>$$O_{ij} \sim \text{Poisson}(\mu_{ij})$$ $$r_{ij} = \exp(\alpha_j + \phi_{ij})$$ $$\log(\mu_{ij}) = \log(E_{ij}) + \alpha_j + \phi_{ij}$$ $$\boldsymbol{\phi}_j \sim \text{Normal}\left(\mathbf{0}, \sigma^2_{\phi_j}(\boldsymbol{D} - \boldsymbol{W})^{-1}\right)$$ $$\alpha_j \sim \text{Normal}(0,1000)$$ $$\frac{1}{\sigma^2_{\phi_j}} \sim \text{Gamma}(0.01, 0.01)$$ $$\boldsymbol{W}_{\boldsymbol{n}\times\boldsymbol{n}} = \begin{cases} w_{ik} = 1 & \text{if } i \sim k \\ w_{ik} = 0 & \text{if } i = k \\ w_{ik} = 0 & \text{otherwise} \end{cases}$$ $$w_{i+} = \sum_k w_{ik}$$ $$\boldsymbol{D}_{n\times n} = \text{diagonal}(w_{1+}, w_{2+}, \dots, w_{n+})$$</td><td>$$O_{ij} \sim \text{Poisson}(\mu_{ij})$$ $$r_{ij} = \exp(\alpha_j + \phi_{ij})$$ $$\log(\mu_{\text{ij}}) = \log(E_{ij}) + \alpha_j + \phi_{ij}$$ $$\begin{bmatrix} \boldsymbol{\phi}_1 \\ \boldsymbol{\phi}_2 \end{bmatrix} \sim \text{Normal}(\mathbf{0}, \boldsymbol{\Sigma} \otimes (\boldsymbol{D} - \boldsymbol{W})^{-1})$$ $$\boldsymbol{\Sigma} = \begin{bmatrix} \sigma^2_{\phi_1} & \rho\sigma_{\phi_1}\sigma_{\phi_2} \\ \rho\sigma_{\phi_1}\sigma_{\phi_2} & \sigma^2_{\phi_2} \end{bmatrix}$$ $$\boldsymbol{\Sigma}^{-1} \sim \text{Wishart}(\boldsymbol{R}, 2)$$ $$\boldsymbol{R} = \begin{bmatrix} 1/5 & 0 \\ 0 & 1/5 \end{bmatrix}$$ $$\alpha_j \sim \text{Normal}(0,1000)$$ $$\boldsymbol{W}_{\boldsymbol{n}\times\boldsymbol{n}} = \begin{cases} w_{ik} = 1 & \text{if } i \sim k \\ w_{ik} = 0 & \text{if } i = k \\ w_{ik} = 0 & \text{otherwise} \end{cases}$$ $$w_{i+} = \sum_k w_{ik}$$ $$\boldsymbol{D}_{n\times n} = \text{diagonal}(w_{1+}, w_{2+}, \dots, w_{n+})$$</td></tr>
<tr><td>In this model, the $\boldsymbol{\phi}_{\boldsymbol{j}}$ vectors are spatially structured random effects Normally distributed with mean zero vector, variance parameters $\sigma^2_{\phi_j}$ and structure matrix $(\boldsymbol{D} - \boldsymbol{W})$, the α_j are intercepts, and the $\boldsymbol{I}$ matrix is an $n \times n$ identity matrix. The $(\boldsymbol{D} - \boldsymbol{W})$ matrix is a structure matrix where **D** is a diagonal matrix of the total number of neighbors of every area, and **W** is a proximity matrix. In the W matrix, the symbol ~ means "neighbor of".</td><td>This time, the $\boldsymbol{\phi}_{\boldsymbol{j}}$ are spatially structured random effects Normally distributed with zero mean vector and variance covariance matrix $\boldsymbol{\Sigma} \otimes (\boldsymbol{D} - \boldsymbol{W})^{-1}$, where $\boldsymbol{\Sigma}$ is the variance-covariance matrix of the $\boldsymbol{\phi}_{\boldsymbol{j}}$, the α_j are intercepts for every disease, and ρ is the correlation parameter in the variance covariance matrix $\boldsymbol{\Sigma}$. The $(\boldsymbol{D} - \boldsymbol{W})$ matrix is a structure matrix where **D** is a diagonal matrix of the total number of neighbors of every area, and **W** is a proximity matrix. In the W matrix, the symbol ~ means "neighbor of". The inverse of the $\boldsymbol{\Sigma}$ variance covariance matrix is assumed Wishart distributed with **R** matrix and 2 degrees of freedom.</td></tr>
</table>

Appendix Table 3. Statistical formulation of joint models of relative risk 5 and 6

Model 5	Model 6
This model accommodates disease-specific IID Normal spatially uncorrelated random effects, and shared-components of spatial clustered patterns of risk for both diseases	This model accommodates disease-specific CAR Normal spatially clustered random effects, and shared-components of spatial clustered patterns of risk for both diseases
$O_{ij} \sim \text{Poisson}(\mu_{ij})$<br>$r_{ij} = \alpha_j + \phi_{ij}$<br>$\log(\mu_{i1}) = \log(E_{i1}) + \alpha_1 + \psi_i/\gamma + \phi_{i1}$<br>$\log(\mu_{i2}) = \log(E_{i2}) + \alpha_2 + \psi_i \times \gamma + \phi_{i2}$<br>$\boldsymbol{\psi} \sim \text{Normal}(\mathbf{0}, \sigma_\psi^2(\boldsymbol{D}-\boldsymbol{W})^{-1})$<br>$\boldsymbol{\phi}_j \sim \text{Normal}\left(\mathbf{0}, \sigma_{\phi_j}^2 \boldsymbol{I}\right)$<br>$\sigma_\psi \sim \text{Uniform}(0,10)$<br>$\sigma_{\phi_j} \sim \text{Uniform}(0,10)$<br>$\alpha_j \sim \text{Normal}(0,1000)$<br>$\gamma \sim \text{Normal}(0,100)$<br>$\boldsymbol{W}_{\boldsymbol{n}\times\boldsymbol{n}} = \begin{cases} w_{ik} = 1 & \text{if } i \sim k \\ w_{ik} = 0 & \text{if } i = k \\ w_{ik} = 0 & \text{otherwise} \end{cases}$<br>$w_{i+} = \sum_k w_{ik}$<br>$\boldsymbol{D}_{n\times n} = \text{diagonal}(w_{1+}, w_{2+}, \dots, w_{n+})$	$O_{ij} \sim \text{Poisson}(\mu_{ij})$<br>$r_{ij} = \alpha_j + \phi_{ij}$<br>$\log(\mu_{i1}) = \log(E_{i1}) + \alpha_1 + \psi_i/\gamma + \phi_{i1}$<br>$\log(\mu_{i2}) = \log(E_{i2}) + \alpha_2 + \psi_i \times \gamma + \phi_{i2}$<br>$\boldsymbol{\psi} \sim \text{Normal}(\mathbf{0}, \sigma_\psi^2(\boldsymbol{D}-\boldsymbol{W})^{-1})$<br>$\boldsymbol{\phi}_j \sim \text{Normal}\left(\mathbf{0}, \sigma_{\phi_j}^2(\boldsymbol{D}-\boldsymbol{W})^{-1}\right)$<br>$\sigma_\psi \sim \text{Uniform}(0,10)$<br>$\sigma_{\phi_j} \sim \text{Uniform}(0,10)$<br>$\alpha_j \sim \text{Normal}(0,1000)$<br>$\gamma \sim \text{Normal}(0,1000)$<br>$\boldsymbol{W}_{\boldsymbol{n}\times\boldsymbol{n}} = \begin{cases} w_{ik} = 1 & \text{if } i \sim k \\ w_{ik} = 0 & \text{if } i = k \\ w_{ik} = 0 & \text{otherwise} \end{cases}$<br>$w_{i+} = \sum_k w_{ik}$<br>$\boldsymbol{D}_{n\times n} = \text{diagonal}(w_{1+}, w_{2+}, \dots, w_{n+})$
In this model, the $\boldsymbol{\phi_j}$ vectors are Normally distributed unstructured spatial random effects for every disease with mean zero vector, $\sigma_{\phi_j}^2$ variance parameters, and identity matrix ***I***. The $\boldsymbol{\psi}$ vector is the spatially structured shared-parameter Normally distributed with zero mean vector, σ_ψ^2 variance parameter, $(\boldsymbol{D}-\boldsymbol{W})$ structure matrix, and γ is a scaling parameter. ***D*** and ***W*** were already defined above.	In this model, the $\boldsymbol{\phi_j}$ vectors are Normally distributed structured spatial random effects for every disease with mean zero vector, $\sigma_{\phi_j}^2$ variance parameters, and identity matrix ***I***. The $\boldsymbol{\psi}$ vector is the spatially structured shared-parameter vector Normally distributed with zero mean vector, σ_ψ^2 variance parameter, $(\boldsymbol{D}-\boldsymbol{W})$ structure matrix, and γ is a scaling parameter. ***D*** and ***W*** were already defined above.

Appendix Table 4. Statistical formulation of joint models of relative risk 7 and 8.

Models 7 and 8
Model 7 accommodates the generalized multivariate conditionally autoregressive (CAR) model (1), where the disease- and area-specific CAR Normal spatially clustered random effects of ZVD are conditioned by the CAR Normal spatially clustered random effects of dengue. Model 8 presents the generalized multivariate CAR model [3], where the disease- and area- CAR Normal spatially clustered random effects of dengue per area are conditioned by the CAR Normal spatially clustered random effects of ZVD.
$O_{ij} \sim \text{Poisson}(\mu_{ij})$<br>$r_{ij} = \alpha_j + \phi_{ij}$<br>$\log(\mu_{ij}) = \log(E_{ij}) + \phi_{ij}$<br>$\boldsymbol{\phi}_1 \mid \boldsymbol{\phi}_2 \sim \text{Normal}(\boldsymbol{\delta_1}, \boldsymbol{\Xi_1})$<br>$\boldsymbol{\delta_1} = \alpha_1 \mathbf{1} + (\eta_0 \boldsymbol{I} + \eta_1 \boldsymbol{W})(\boldsymbol{\phi}_2 - \alpha_2 \mathbf{1})$<br>$\Xi_1 = \sigma_1^2 (\boldsymbol{D} - \kappa_1 \boldsymbol{W})^{-1}$<br>$\boldsymbol{\phi_2} \sim \text{Normal}(\boldsymbol{\delta_2}, \boldsymbol{\Xi_2})$<br>$\boldsymbol{\delta_2} = \alpha_2 \mathbf{1}$<br>$\boldsymbol{\Xi_2} = \sigma_1^2 (\boldsymbol{D} - \kappa_2 \boldsymbol{W})^{-1}$<br>$\alpha_j \sim \text{Normal}(0,100)$<br>$\eta_0, \eta_1 \sim \text{Normal}(0,10)$<br>$\sigma_j \sim \text{Uniform}(0,10)$<br>$\kappa_j \sim \text{Uniform}(0,0.99)$<br>$\boldsymbol{W_{n\times n}} = \begin{cases} w_{ik} = 1 & \text{if } i \sim k \\ w_{ik} = 0 & \text{if } i = k \\ w_{ik} = 0 & \text{otherwise} \end{cases}$<br>$w_{i+} = \sum_k w_{ik}$<br>$\boldsymbol{D}_{n\times n} = \text{diagonal}(w_{1+}, w_{2+}, \dots, w_{n+})$
The parameters in Models 7 and 8 are: ϕ_{ij} parameters are spatially structured random effects, where the $\boldsymbol{\phi_1}$ random effects vector for disease 1 is conditioned on the $\boldsymbol{\phi_2}$ random effects vector for disease 2. The ϕ_{ij} parameters are Normally distributed with $\boldsymbol{\delta_j}$ mean vector and Ξ_j variance covariance matrix. The $\boldsymbol{\delta_1}$ mean vector depends of the parameters α_1, η_0, η_1, and α_2, the vector of one's **1**, the identity matrix ***I***, and the proximity matrix ***W***, while the covariance matrix $\boldsymbol{\Xi_1}$ depends on the variance σ_1^2, the κ_1 parameter, and the diagonal matrix **D** and the proximity matrix **W**. In contrast, the $\boldsymbol{\delta_2}$ mean vector only depends of the α_1 parameter, while the $\boldsymbol{\Xi_2}$ variance covariance matrix depends of the variance σ_2^2, the κ_2, and the diagonal matrix **D** and the proximity matrix **W**.

Appendix Table 5. Predictive check on over dispersion by the joint Models 1 to 8, for the department of Santander and the city of Bucaramanga.

	Bucaramanga			Santander	
Model	Dengue	Zika	Model	Dengue	Zika
1	0.71	0.78	1	0.50	0.50
2	0.51	0.88	2	0.51	0.50
3	0.62	0.68	3	0.51	0.49
4	0.43	0.81	4	0.50	0.49
5	0.70	0.83	5	0.51	0.50
6	0.41	0.82	6	0.51	0.50
7	0.39	0.86	7	0.51	0.50
8	0.48	0.81	8	0.50	0.49

Appendix Table 6. Spearman correlation coefficients for the posterior mean of the fitted values and observed values for the joint Models 1 to 8 of relative risk

	Santander			Bucaramanga	
Model	Dengue	Zika	Model	Dengue	Zika
1	0.995	0.994	1	0.962	0.972
2	0.995	0.987	2	0.938	0.958
3	0.995	0.992	3	0.944	0.957
4	0.992	0.976	4	0.925	0.945
5	0.995	0.984	5	0.932	0.949
6	0.994	0.980	6	0.922	0.945
7	0.994	0.988	7	0.928	0.952
8	0.995	0.984	8	0.929	0.947

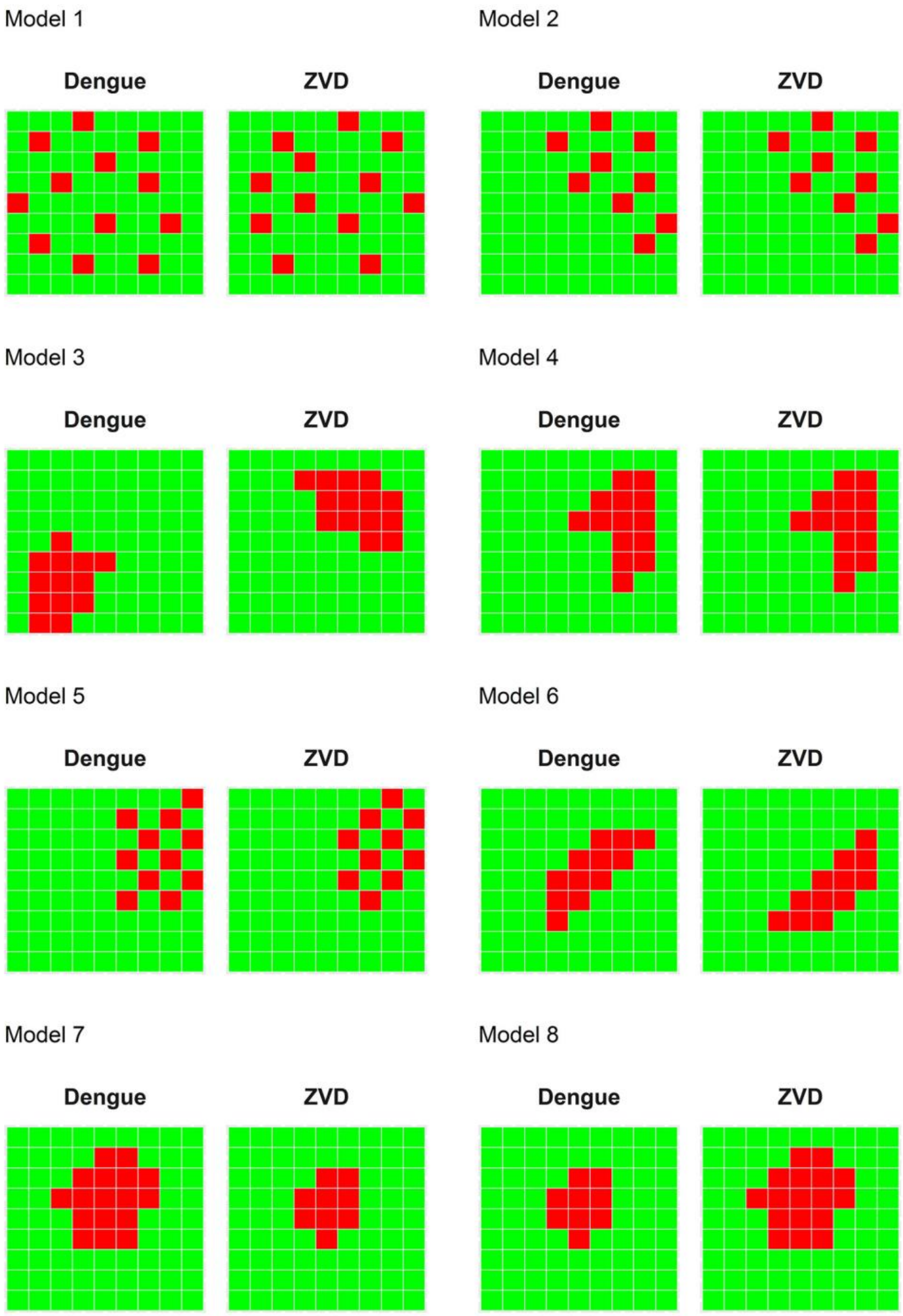

Appendix Figure 1. Spatial patterns represented by the joint models of relative 1 to 8 for dengue and ZVD.

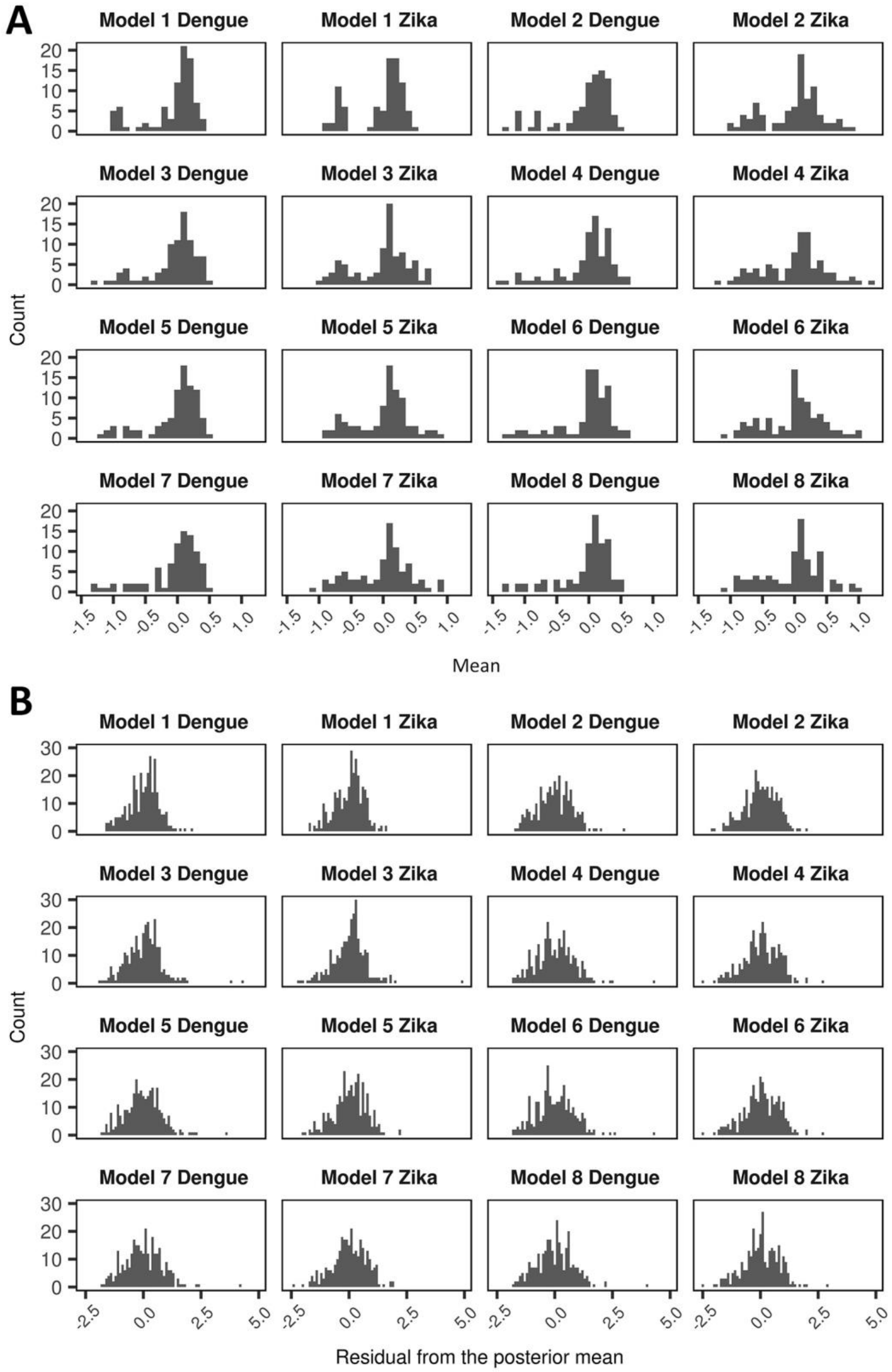

Appendix Figure 2. Histogram of the residuals for the joint Models 1 to 8 of (A) the department of Santander, and (B) the city of Bucaramanga.

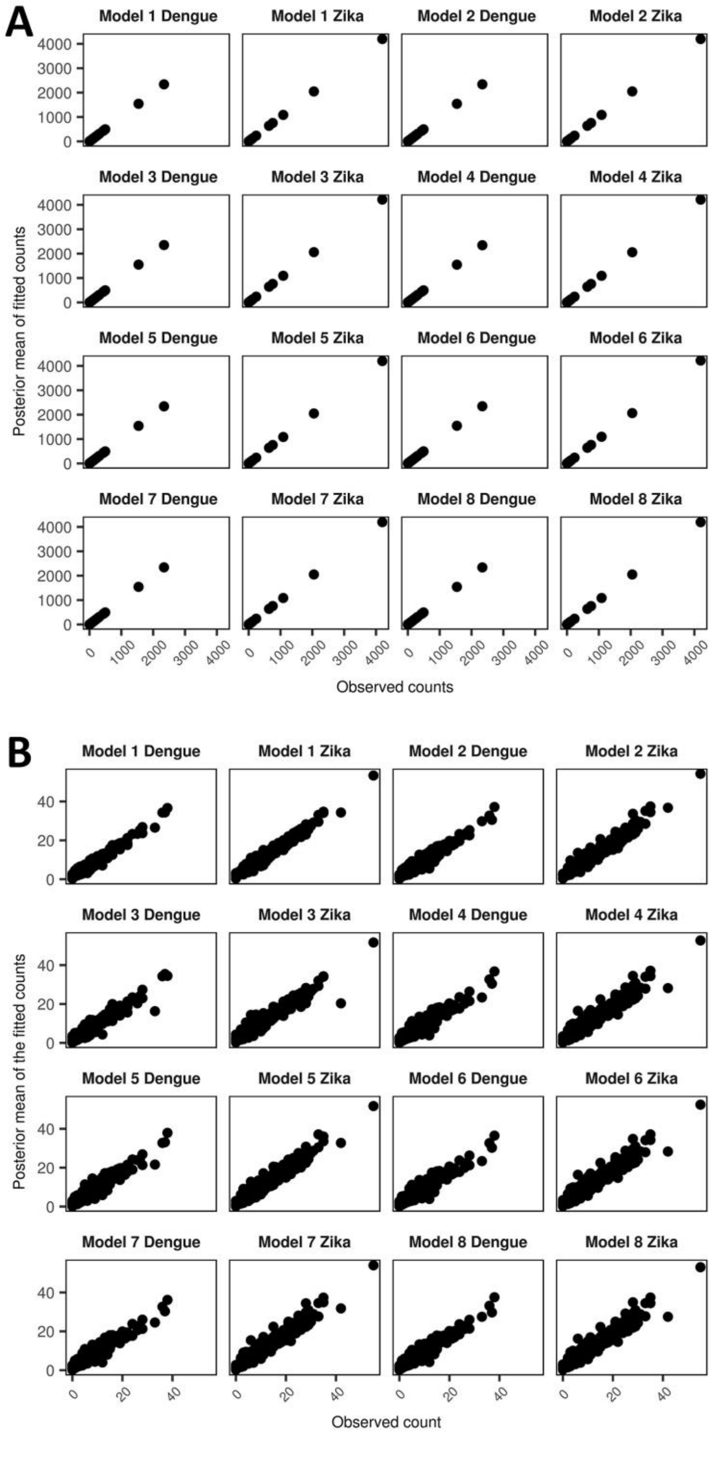

Appendix Figure 3. Scatter plot for the posterior mean of the fitted values versus the observed values of dengue and ZVD from the joint Models 1 to 8, (A) department of Santander, and (B) city of Bucaramanga.
